# Identifying Genes that Affect Differentiation of Human Neural Stem Cells and Myelination of Mature Oligodendrocytes

**DOI:** 10.1007/s10571-022-01313-5

**Published:** 2022-12-22

**Authors:** Dou Ye, Qian Wang, Yinxiang Yang, Bingyu Chen, Fan Zhang, Zhaoyan Wang, Zuo Luan

**Affiliations:** 1 Medical School of Chinese PLA, Beijing, China; 2 Department of Pediatrics, the sixth Medical Centre, Chinese PLA General Hospital, Beijing, China; 3 Guangxi Medical University, Nanning, Guangxi China

**Keywords:** OPCs, Oligodendrocytes lines, The differentiation of NSCs, The maturation of OLs, GO, KEGG, RNA-Seq

## Abstract

Human neural stem cells (NSCs) are self-renewing, multipotent cells of the central nervous system (CNS). They are characterized by their ability to differentiate into a range of cells, including oligodendrocytes (OLs), neurons, and astrocytes, depending on exogenous stimuli. An efficient and easy directional differentiation method was developed for obtaining large quantities of high-quality of human OL progenitor cells (OPCs) and OLs from NSCs. RNA sequencing, immunofluorescence staining, flow cytometry, western blot, label-free proteomic sequencing, and qPCR were performed in OL lines differentiated from NSC lines. The changes in the positive rate of typical proteins were analyzed expressed by NSCs, neurons, astrocytes, OPCs, and OLs. We assessed Gene Ontology (GO) enrichment and Kyoto Encyclopedia of Genes and Genomes (KEGG) pathways of differentially expressed (DE) messenger RNAs (mRNAs) related to the differentiation of NSCs and the maturation of OLs. The percentage of NSCs differentiated into neurons, astrocytes, and OLs was 82.13%, 80.19%, and 90.15%, respectively. We found that nestin, PAX6, Musashi, and vimentin were highly expressed in NSCs; PDGFR-α, A2B5, NG2, OLIG2, SOX10, and NKX2-2 were highly expressed in OPCs; and CNP, GALC, PLP1, and MBP were highly expressed in OLs. RNA sequencing, western blot and qPCR revealed that ERBB4 and SORL1 gradually increased during NSC–OL differentiation. In conclusion, NSCs can differentiate into neurons, astrocytes, and OLs efficiently. PDGFR-α, APC, ID4, PLLP, and other markers were related to NSC differentiation and OL maturation. Moreover, we refined a screening method for ERBB4 and SORL1, which may underlie NSC differentiation and OL maturation.

**Graphical Abstract:**

Potential unreported genes and proteins may regulate differentiation of human neural stem cells into oligodendrocyte lineage. Neural stem cells (NSCs) can differentiate into neurons, astrocytes, and oligodendrocyte (OLs) efficiently. By analyzing the DE mRNAs and proteins of NSCs and OLs lineage, we could identify reported markers and unreported markers of ERBB4 and SORL1 that may underlie regulate NSC differentiation and OL maturation.

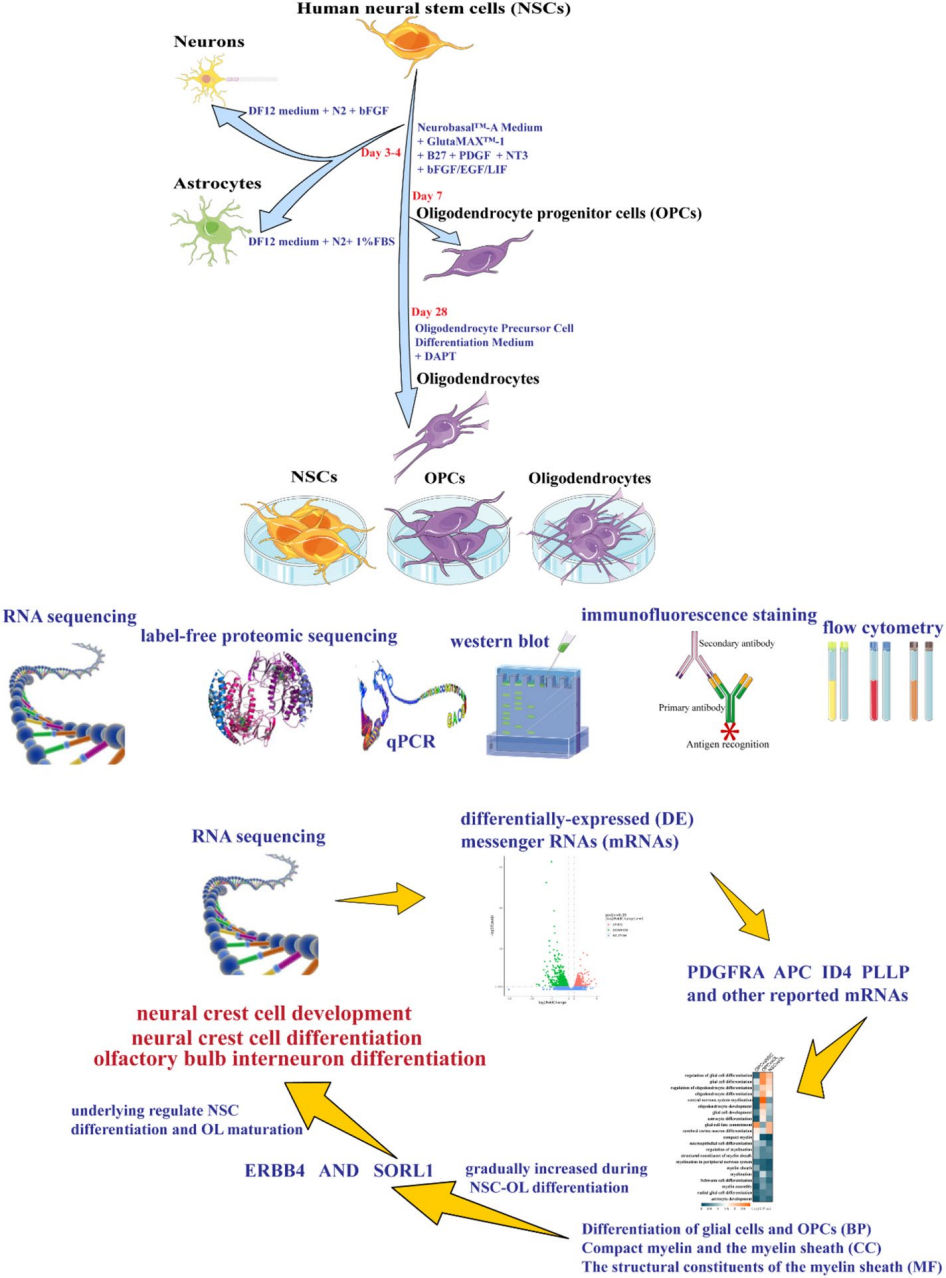

**Supplementary Information:**

The online version contains supplementary material available at 10.1007/s10571-022-01313-5.

## Background

The oligodendrocyte (OL), a type of glial cell, is essential for the generation of the myelin sheath surrounding axonal processes in the mammalian central nervous system (CNS) (Biswas et al. 2019; Gargareta et al. 2022). Myelin is critical for axonal integrity, nerve conduction, and cell survival. OL progenitor cells (OPC) pass through multiple developmental stages before maturing into myelinating OLs (Barateiro and Fernandes 2014; Kang and Yao 2022; Li et al. 2022). Although the lineage commitment of OLs is highly conserved between humans and mice, the phenological timeline of human brain development remains fragmented (Chanoumidou et al. 2020). For example, gliogenesis begins in the second trimester of embryonic development and extends into adulthood (Jakovcevski et al. 2009; Semple et al. 2013; Dietz et al. 2016), while in rodents it begins postnatally (Rivers et al. 2008). To elucidate the processes related to the maturation of OLs, it is necessary to identify differences in messenger RNA (mRNA), proteins, and cell markers between stages of differentiation, and to characterize potential differentially expressed (DE) mRNA, gene ontology (GO) enrichments, and Kyoto Encyclopedia of Genes and Genomes (KEGG) pathways that induce differentiation and myelination.

Conventional sources for generating OPCs include human embryonic stem cells (hESCs) (Otsu et al. 2021), human induced pluripotent stem cells (hiPSCs) (Llorente et al. 2021), and human fetal brain tissue (Monaco et al. 2012). Neural stem cells (NSCs) generated from hESCs and hiPSCs have supplemented our knowledge of human OL and glial cell development (Wegscheid et al. 2021; Statoulla et al. 2021). However, the clinical application of NSCs, as well as OLs, derived from hESCs and hiPSCs is limited by low differentiation rates (Wang et al. 2013). Deriving OPCs from hESCs and hiPSCs is also time-consuming (Wang et al. 2013), notwithstanding the risk of tumorigenicity (Wang et al. 2013). Furthermore, conventional strategies for generating OPCs and human OLs are applied without the establishment or amplification of stem/progenitor cells. Additionally, OPCs induced by NSCs derived from human fetal brain tissue have the advantages of low tumorigenicity risk, efficient preparation, and easy transformation (Dietz et al. 2016; Azari 2022).

Luan et al. (Wang et al. 2015) have successfully directed human fetal NSCs into highly pure OPCs using a cocktail of basic fibroblast growth factor, platelet-derived growth factor, and neurotrophic factor-3. Of these cells, 80–90% expressed specific OPC markers, such as SOX10 and PDGFR-α. Based on these results, using a culture system of NSCs established by Luan et al., we differentiated mature OLs on the basis of OPCs in our study. We aimed to develop a systematic method for amplifying stem/progenitor cells and differentiating OLs from OPCs. We described morphological changes, protein differences, and functional and pathway differences in NSCs, OPCs, and OLs. Moreover, we aimed to develop a method for identifying key mRNAs and pathways related to the differentiation and myelination of OLs. RNA sequencing (RNA-seq) was used to identify DE mRNA profiles in NSCs, OPCs, and OLs. We also conducted GO and KEGG enrichment analyses, constructed a gene–gene interaction network, and screened pivotal mRNA.

## Methods

### Culture of NSCs, Neuron, Astrocyte, OPCs, and OLs

NSCs were prepared in the pediatric laboratory of the Sixth Medical Centre of PLA General Hospital, Beijing, China, on September 2021, using previously established methods of cultivation (Wang et al. 2015). NSC line has recently been authenticated and by National Institutes for Food and Drug Control of China (Report Number: SH20220032). NSCs were seeded into T75 or T225 cell culture bottles. NSCs were incubated for 10 days on a self-made medium at 37 °C in a humidified atmosphere with 5.0% CO_2_ and used for induction. The neuro-spheres were dissociated into single cells and re-suspended in neuronal differentiation medium which contains DMEM/F-12 (DF-12, Cat. #11330032, Gibco, Thermo Fisher Scientific, Waltham, Massachusetts, USA) medium mixed with N-2 supplements (Cat. # 17502048, Invitrogen, Thermo Fisher Scientific, Waltham, Massachusetts, USA) and recombinant Human FGF-basic (bFGF; Cat. #100-18C, Pepro Tech Inc, New Jersey, USA) for neurons, and in astrocyte differentiation medium which contains DF12 medium mixed with N2 and 1%FBS (Cat. #10099141, Gibco, Thermo Fisher Scientific, Waltham, Massachusetts, USA) for astrocyte. For neurons and astrocytes, forty thousand cells /well were inoculated in a 24-well plate (Cat. #3524, Coring, Arkansas, USA). Half of the fluid was changed at intervals of 3–4 days. The single cells were re-suspended in OPC differentiation medium which contains Neurobasal™-A Medium mixed with 30 μM putrescine, 5 μg/mL transferrin, 2 mM GlutaMAX™-1, 5 μg/mL insulin, 10 nM progesterone, 30 μM putrescine, 15 nM sodium selenium, 5 μg/mL Heparin, 5 μM lactate, 2% B27, 10 ng/mL platelet-derived growth factor (PDGF), 10 ng/mL neurotrophic factor-3 (NT3), 5 ng/mL bFGF, 20 ng/mL epidermal growth factor (EGF), 10 ng/mL leukemia inhibitory factor (LIF), and 100 U/ml penicillin and streptomycin (materials and reagents provided in Supplemental Table 1) and inoculated on a 6-well plate (Cat. #3516, Coring, Arkansas, USA) with 200,000 OPCs /well. Half the medium of OPCs was replenished every 3 days. The cells slowly migrated out of the neuro-spheres. When the cells reached 90% confluence, they were passaged and incubated on 6-well plates at a density of 200,000 cells/well for 7 days. The OPCs were passaged to P3 and prepared for differentiation into OLs and amplification thereof. OLs were cultured in a OLs differentiation medium which contains DMEM/F12 mixed with 1% Oligodendrocyte Precursor Cell Differentiation Medium (OPCDM) 1 μM DAPT, and 100 U/ml penicillin and streptomycin (materials and reagents provided in Supplemental Table 2) on a 24-well plate (Cat. #3524, Coring, Arkansas, USA) with 20,000 cells /well or a 6-well plate with 200,000 cells /well which were pre-coated by Poly-l-ornithine hydrobromide (PLO; Cat. # P3655, Sigma-Aldrich, St. Louis, MO, USA) and laminin (LN; Cat. #2399234, Gibco, New York, USA) for 21 days. Half the medium of OLs was replenished every 5 days. Morphology in widefield of NSCs, OPCs, and OLs were observed via microscopy (IX-70, Olympus Corporation, Tokyo, Japan) at room temperature and were taken by an Olympus with a 20 × 0.40 PHC and 40 × 0.55 PH2 macro lens. Digital images were captured by DP2-BSW software (ver.2.1, Olympus Corporation, Tokyo, Japan). NSCs, OPCs, and OLs were prepared for qPCR, flow cytometry, RNA-seq, label-free proteomic sequencing, and western blotting.

### Immunofluorescence Staining

Appropriately sized neuro-spheres were cultured for 1, 2, and 5 days, and the OPCs were passaged 3–5 times, after which they were induced and differentiated into OLs for 10, 15, and 21 days. The neurons and astrocytes were cultured for 7 days. The neuro-spheres or their digested single cell, neurons, astrocytes, and OPCs were then inoculated into 8-well-cell culture slides (Cat. #072108, BIOLOGIX, Shangdong, China) pre-coated with fibronectin human plasma (FN; Cat. #SLCG9672, Sigma-Aldrich, St. Louis, MO, USA) and LN in DMEM-F12 supplemented with 2% B27 (Cat. #17504-044, Gibco, New York, USA), then cultured for 24–48 h and fixed with 4% paraformaldehyde solution in PBS (PFA, Cat. #P1110, Solarbio, Beijing, China) for immunofluorescence staining. OLs for 10, 15, and 21 days were directly fixed with 4% paraformaldehyde solution in PBS in 24-well plates. The NSCs were identified using monoclonal mouse anti-nestin (1:100; Cat. #ab6320, Abcam, Cambridge, Cambridgeshire, UK, RRID: AB_308832) antibodies. Mouse anti-A2B5 (1:50; Cat. #MAB1416, R&D Systems, Minneapolis, MN, USA, RRID: AB_357687), rabbit anti-OLIG2 (1:300; Cat. #AB9610, Millipore, MA, USA, RRID: AB_570666), mouse anti-SOX10 (1:300; Cat. #MAB2864, R&D Systems, Minneapolis, MN, USA, RRID: AB_2195180), rabbit anti-NG2 (1:100; Cat. #ab83178, Abcam, Cambridge, UK, RRID: AB_10672215), and rabbit anti-PDGFR-α (1:800; Cat. #C5241, Cell Signaling Technology, Boston, MA, USA, RRID: AB_10692773) were used to detect OPCs. Oligodendrocytes were identified using anti-GALC (1:100; Cat. #MAB342, Millipore, MA, USA, RRID: AB_94857), anti-PLP (1:50; Cat. #ab28486, Abcam, Cambridge, UK, RRID: AB_776593), and anti-MBP (1:100; Cat. #ab7349, Abcam, Cambridge, UK, RRID: AB_305869) antibodies. NSCs, OPCs, and OLs were also identified using rabbit anti-ERBB4 (1:100; Cat. #19943-1-AP, Proteintech, Chicago, USA, RRID: AB_10646486) and rabbit anti-SORL1 (1:800; Cat. #79322, Cell Signaling Technology, Boston, USA, RRID: AB_2799927). Alexa 488-conjugated donkey anti-mouse (1:500; Cat. # ab150105, Abcam, Cambridge, UK, RRID: AB_2732856), Alexa 594-conjugated donkey anti- rabbit (1:500; Cat. # ab150076, Abcam, Cambridge, UK, RRID: AB_2782993), and Alexa 488-conjugated donkey anti-rabbit (1:500; Cat. # ab150153, Abcam, Cambridge, UK, RRID: AB_2737355) secondary antibodies were used, respectively, for immunocytochemistry. Cell nuclei were labeled with 4′,6-diamidino-2-phenylindole (DAPI; 1:20; Cat. #28718-90-3, Sigma-Aldrich, St. Louis, MO, USA). Images of immunofluorescence staining were captured using a digital confocal laser scanning microscope (20 × and 40 × for detailed imaging, room temperature, FV1000-D, OLYMPUS, Tokyo, Japan) and fluorescence microscopy (20 × for quantification, IX-70, Olympus Corporation, Tokyo, Japan). For quantification, the positive rate was calculated from at least three random visual fields for at least three images. Positive rate of markers = N1/N2, where N1 refers to the number of corresponding antibody-labeled cells and N2 refers to the total number of DAPI-labeled cells.

### Flow Cytometry of NSCs and OPCs

As OL cultures require continuous adherent culturing and cannot be passaged, the resulting cells are difficult to digest, and the viability of digested cells is low. This makes it difficult to meet the requirements of flow cytometry. For the analysis of protein expression in NSCs and OPCs, the cells were digested and washed once with buffer and centrifuged at 2000×*g* for 5 min at 4 ℃. Human TruStain FcX™ Fc Receptor Blocking Solution (Fc; Cat. #422302, Biolegend, California, USA, RRID: AB_2818986) was incubated for 10 min at 25 °C. NSCs were stained with PE nestin mouse anti-human (Cat. #561230, BD Biosciences, Franklin Lake, NJ, USA, RRID: AB_10562398), PE Musashi mouse anti-human (Cat. #561468, BD Biosciences, Franklin Lake, NJ, USA, RRID: AB_10689629), FITC vimentin mouse anti-human (Cat. #562338, BD Biosciences, Franklin Lake, NJ, USA, RRID: AB_10896994), and APC PAX6 mouse anti-human (Cat. #562249, BD Biosciences, Franklin Lake, NJ, USA, RRID: AB_11152956) antibodies. OPCs were stained with BV421 PDGFR-α mouse anti-human (Cat. #562799, BD Biosciences, Franklin Lake, NJ, USA, RRID: AB_2737804), FITC SOX10 mouse anti-human (Cat. #NBP2-47709F, NOVUS Biologicals, Colorado, USA, RRID: AB_2923505), PE A2B5 mouse anti-human (Cat. #130-093-581, Miltenyi Biotec, Bergisch-Gladbach, Germany, RRID: AB_10830711), and FITC OLIG2 mouse anti-human (Cat. #AB9610, Millipore, MA, USA, RRID: AB_570666) antibodies for 30 min at 4 °C. After staining, NSCs and OPCs were washed once with buffer and centrifuged at 2000×*g* for 5 min at 4 °C. Cells were analyzed using a FlowSight® imaging flow cytometer (Amnis®, part of EMD Millipore). Cell debris and dead cells were identified and removed based on the aspect ratio and area of the cells. The data were analyzed using FlowJo software v10 (FlowJo, Ashland, OR, USA).

### Western Blot

NSCs, OPCs, and OLs were homogenized on ice with RIPA lysis buffer (Cat. # HX1862-1, Huaxingbio, Beijing, China) containing protease and phosphatase inhibitors. The homogenates were centrifuged at 15,000×*g* at 4 °C for 10 min, and the supernatants were collected. Approximately 20 μg of total protein was extracted, resolved using equal amounts of SDS-PAGE (Cat. #Ba1012, Baiqiandu, Wuhan, China), and transferred to a PVDF membrane (Cat. #IPVH00010, Millipore, MA, USA). Membranes were blocked with 5% milk for 1 h and then probed with primary antibodies (Supplementary Table 3) at 4 °C overnight according to manufacturer’s instructions. They were then washed three times with TBST (Cat. #Ba1024, Baiqiandu, Wuhan, China) and incubated with secondary antibodies, goat anti-rabbit IgG H&L (Cat. #ab205718, Abcam, Cambridge, UK, RRID: AB_2819160) and goat anti-mouse IgG H&L (Cat. #ab205719, Abcam, Cambridge, UK, RRID: AB_2755049), for 60 min at room temperature. The membranes were then imaged with an EPSON scanner (L1548, Nagano Prefecture, Japan). The optical density of the membranes was analyzed by the ImageJ software processing system.

### Label-Free Proteomic Sequencing

The samples were subjected to SDS-PAGE electrophoresis. Proteins were digested by incubation with trypsin (trypsin: protein = 1:50; Cat. #V5280, Promega, Wisconsin, USA) at 37 °C overnight, which was terminated with 50 µL 0.1% formic acid (Cat. #T94318, Sigma-Aldrich, Missouri, USA) and eluted with 70% acetonitrile (ACN, Cat. #34851, J.T. Baker, New Jersey, USA). High performance liquid chromatography (HPLC, RIGOL L-3000, Beijing China) was used for protein identification. Any digested peptides were separated before placing the protein samples in the mass spectrometer. Data were analyzed using the Proteome Discoverer suite (version 2.4, Thermo Fisher Scientific, Waltham, Massachusetts, USA). MS2 spectra were searched against the UniProtKB (https://www.uniprot.org) human proteome database containing Swiss-Prot (https://www.sib.swiss/swiss-prot) human reference protein sequences. The Sequest HT search engine was used, and parameters were specified as follows: fully tryptic specificity, maximum of two missed cleavages, minimum peptide length of 6, fixed carbamidomethylation of cysteine residues (+ 57.02146 Da), variable modifications for oxidation of methionine residues (+ 15.99492 Da), a precursor mass tolerance of 15 ppm, and a fragment mass tolerance of 0.02 Da for MS2 spectra collected in the Orbitrap. Percolator was used to filter peptide spectral matches and peptides to a false discovery rate (FDR) of less than 1%. As default, the top matching protein or ‘master protein’ is the protein with the largest number of unique peptides and with the smallest value in the percent peptide coverage (that is, the longest protein).

### qPCR

RNA was extracted using an RNA prep Pure Cell/Bacteria Kit (Cat. #DP430, Tiangen, Beijing, China), treated with deoxyribonuclease I (DNase, 1500 U, Cat. #RT411, Tiangen) for 30 min at 37 °C to avoid genomic contamination, and the quantity and quality of total RNA were assessed. Only those samples that exhibited no DNA contamination and showed a clear gel image were used for subsequent experiments. Briefly, 15 ng of total RNA was transcribed into complementary DNA (cDNA) using Multi-Scribe Reverse Transcriptase (RR036A PrimeScript RT Master Mix, Takara, Kusatsu, Japan). qPCR was performed using a Chromo4 Real-Time PCR Detection System (Cat. #3591590G, Bio-Rad Laboratories, Hercules, CA, USA). Quantification of relative gene expression was conducted according to the 2^−ΔΔ*Ct*^ method. The housekeeping gene for standardizations was β-actin. The primer sequences are listed in Supplementary Table 4.

### RNA-seq

We performed RNA-seq on successively passaged neuro-spheres (NSCs; P10–P12), successively passaged OPCs (P0–P5), and differentiated OLs (cultured for 10, 15, and 21 days). RNA-seq was performed at the Beijing Novo-gene Bioinformatics Technology Co. (Beijing, China). Bioanalyzer 2100 system (Agilent Technologies, Santa Clara, CA, USA) and an RNA Nano 6000 Assay Kit were used to evaluate RNA integrity. Total RNA was used as input for RNA sample preparation and the PCR product was purified with a NanoPhotometer® (Cat. #NP80, Implen, Munich, Germany) to obtain an RNA library. After the library was qualified, different libraries were sequenced using the Illumina NovaSeq 6000. Image data were converted into sequence data (reads) by CASAVA base recognition. Based on clean high-quality data, downstream analyses were performed. Counts were used as the number of reads mapped to each gene. Based on the length and number of reads for each gene, the FPKM was then calculated.

### GO and KEGG

Differential analysis of two groups (three biological replicates per group) was performed using the DESeq2 package (v1.20.0) in R (The R Foundation, Vienna, Austria). *P-*values were adjusted using the Benjamini–Hochberg approach for controlling false discovery rate. The thresholds for significant DE were *P*-adj ≤ 0.05 and log2[fold change] ≥ 1. The genes that met this threshold were gradually increased when comparing NSCs with OPCs, and OPCs with OLs, to identify previously unrecognized mRNA involved in differentiation. GO enrichment analysis of DE mRNAs was performed using the clusterProfiler R package (v3.8.1); GO terms with adjusted *P-*values of < 0.05 and increase ≥ 1 were considered significantly enriched by DE mRNAs. For the KEGG pathway analysis, we used large-scale molecular datasets generated by genome sequencing and other high-throughput experimental technologies (http://www.genome.jp/kegg/), and assessed the statistical enrichment of DE genes in KEGG pathways using the clusterProfiler R package (v3.8.1). Available interaction networks for DE mRNA, DE proteins, GO enrichment, and KEGG pathways were used to predict the interactions of target gene sequences. The selected GO or KEGG sequences were then mapped using Cytoscape software (v3.5.1, Boston, MA, USA).

### Statistical Analysis

Every individual in the chosen samples should have an equal chance to be included in the experiment. Data were analyzed by GraphPad Prism v8 (GraphPad Software, San Diego, CA, USA, www.graphpad.com) and SPSS version 26.0 (IBM SPSS, Chicago, IL, USA). Data are expressed as the mean ± standard deviation (SD). We used SPSS software to perform normality test and variance homogeneity test on the samples. Two samples that met the above conditions were subjected to independent sample Student's *t *tests, and those that did not meet the above two conditions were subjected to one-way analysis of variance (ANOVA) test; For three samples, when they met the homogeneity of variances, use the LSD test for post hoc multiple comparison, and if they did not meet the homogeneity of variances test, they were subjected to the Tamhini test. Differences were considered to be statistically significant at *P* < 0.05.

## Results

### Establishment of a Purified NSC Cell Line

**Fig. 1:**
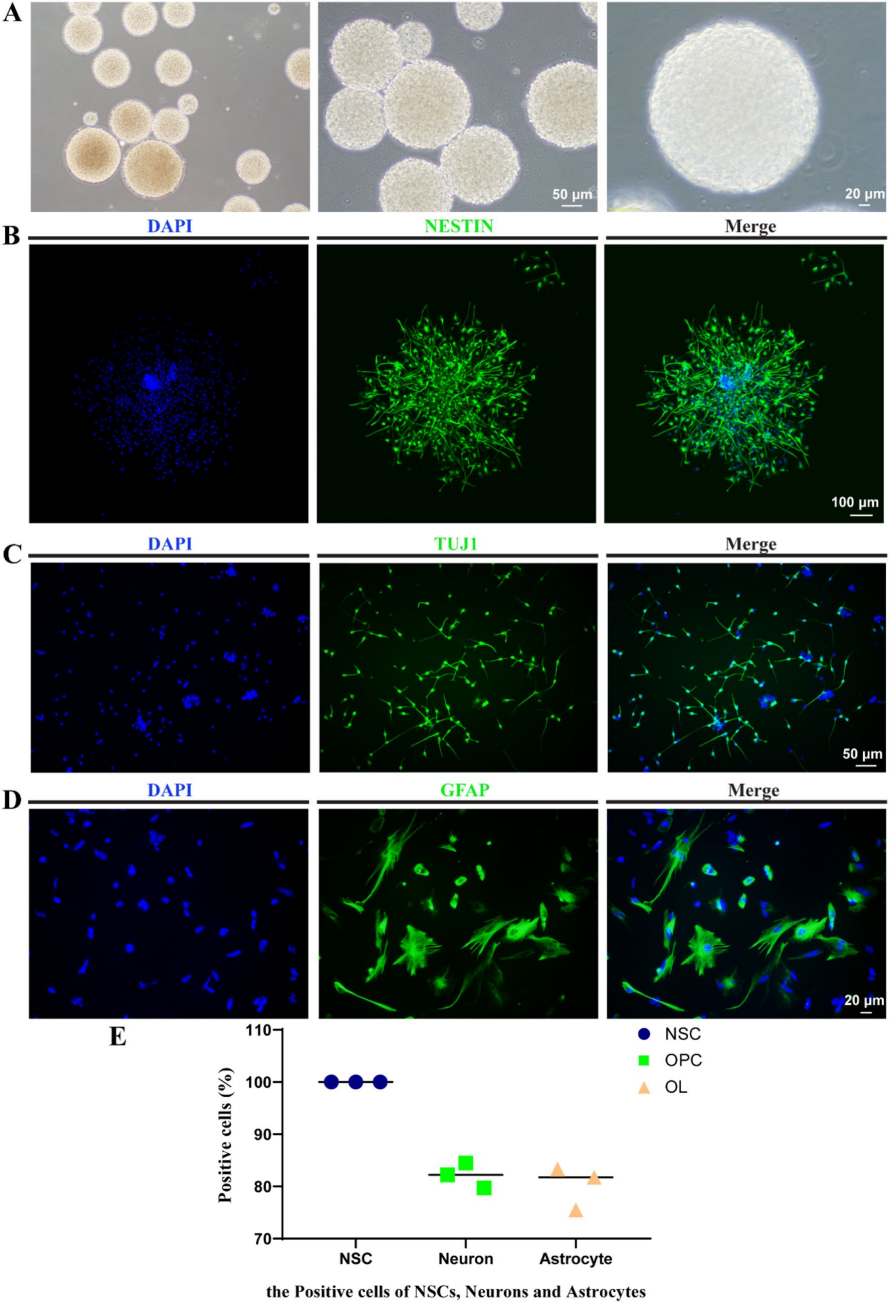
Identification of NSCs from human fetal brain tissue. **a** Morphology of NSCs observed via phase-contrast microscopy; scale bars = 50 or 20 μm. **b** nestin in NSCs as confirmed by immunofluorescence assay; scale bar = 50 μm. **c**, **d** Protein expressions of TUJ1 and GFAP in neurons and astrocytes, respectively, as confirmed by immunofluorescence assay; scale bar = 20 μm. **e** Proportions of nestin^+^ NSCs, TUJ1^+^ neurons, and GFAP^+^ astrocytes analyzed by immunofluorescence staining, NSCs directional differentiate into neurons and astrocytes with high efficiency

NSCs were cultured in suspension and grown as “neuro-spheres.” After 10 days of passage, NSCs appeared spherical, had a good refractive index, and exhibited minute protrusions around the spheroids; the sizes of the neuro-spheres varied, with an average diameter of 200–250 μm (Fig. 1a). Immunofluorescence staining showed a positive result for the NSC-specific antigen, nestin (Fig. 1b). Immunofluorescence staining of directed differentiated cells from NSCs showed that neurons (Fig. 1c) were positive for TUJ1 (median of positive rate: 82.13%) (Fig. 1e), astrocytes (Fig. 1d) for GFAP (mean of positive rate: 80.19%) (Fig. 1e), and OPCs for PDGFR-α (mean of positive rate: 90.15%) (Fig. 3b). Immunofluorescence staining was performed here to identify the characteristics of differentiating directly into neurons, astrocytes, and oligodendrocytes to verify the three-lineage differentiation potential of NSCs in our study.

### Differentiation of NSCs to OLs

**Fig. 2:**
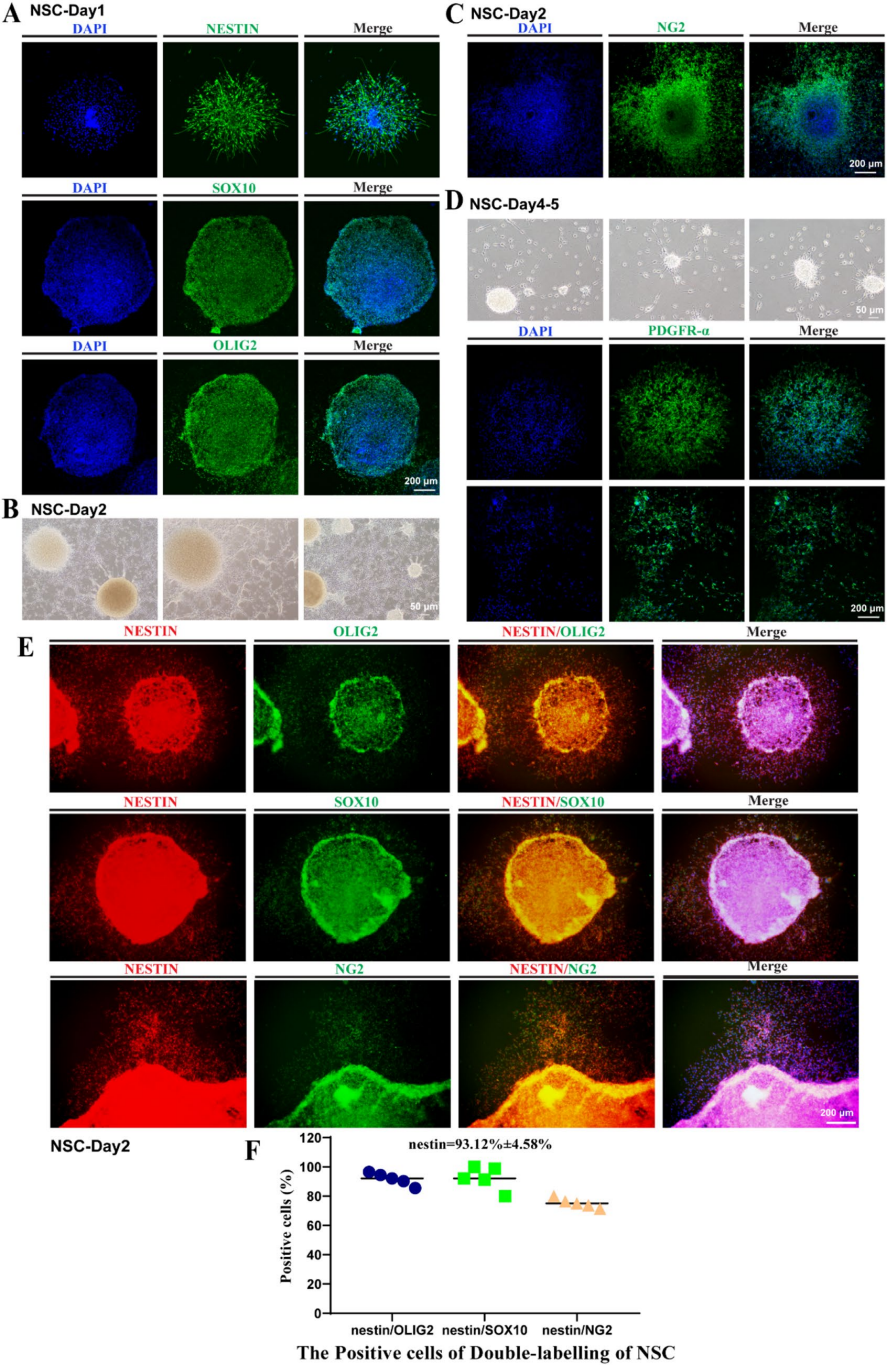
Differentiation of NSCs to OPCs. **a** Protein expressions of nestin, SOX10, and OLIG2 in NSCs (day 1) as confirmed by immunofluorescence assay; scale bar = 200 μm. **b** Morphologies of NSCs observed on day 2; scale bar = 50 μm. **c** NG2^+^ cells as observed by immunofluorescence assay; scale bar = 200 μm. **d** Morphologies of NSCs observed by phase-contrast microscopy and PDGFRA on days 4–5 as confirmed by immunofluorescence assay; scale bar = 50 and 200 μm. **e** Double immunofluorescence staining with nestin/OLIG2, nestin/SOX10, and nestin/NG2 on neuro-spheres of NSCs, scale bar = 200 μm. **f** Proportions of nestin^+^/OLIG2^+^, nestin^+^/SOX10^+^, and nestin^+^/NG2^+^ NSCs digested from neuro-spheres analyzed by double immunofluorescence staining. Means ± SD from three independent experiments represented. For data that met the normality test, the positive rate was shown as the mean, otherwise it is shown as the median. Two samples above that met the above conditions were subjected to independent sample Student's *t* tests Exact *P* values have been reported. Exact *P* values have been reported

On the first day of differentiation of neuro-spheres, aside from the neuroepithelial stem cell protein, nestin, and the OL lineage markers, OLIG2 and SOX10, were also expressed in NSCs (Fig. 2a, d). During oligodendrocyte development, OLIG2 is instrumental for cell fate specification, is one of factor required for OPC production; OLIG2 inactivation leads to a reduced production of OPCs in most CNS regions. Whereas SOX10 loss has no effect during determination but results in failure in terminal cell differentiation; SOX10 is necessary for cell differentiation and maturation, inducing key elements of the regulatory network of differentiating oligodendrocytes. Combining the identification of OLIG2 in Western blot of NSCs which indicated that the neuro-spheres had the potential to differentiate into OL cell lines (El Waly et al. 2018). On the second day, OL precursor cells slowly migrated out of the neuro-spheres (Fig. 2b). Moreover, clusters of cells expressing NG2 were observed (Fig. 2c), and early OPCs began to form. For the fate of NSCs differentiated into oligodendrocytes, we performed multiple marker staining with nestin/OLIG2, nestin/SOX10, and nestin/NG2. Since neural stem cells are cultured in the form of suspended neuro-spheres (Fig. 2e), we digested the neuro-sphere into a single cell for immunofluorescence staining to calculate the positive rate of multiple markers. The positive rate of nestin was 93.12% ± 4.58%, and the positive rates of OLIG2, SOX10, and NG2 double immunofluorescence stained with nestin were 91.19% ± 3.78%, 75.34% ± 2.86%, and 92.45% ± 7.13%, respectively (Fig. 2f). On the fourth and fifth days of differentiation, OPCs stretched to the edges of the neuro-spheres, adhered stably, rapidly divided, and then migrated outwards. Moreover, bipolar, bead-like OPCs appeared (Fig. 2d, e), at which point the expression of PDGFR-α (mean: 90.15%) was observed (Fig. 2d, e). Cells at the edge of the neuro-sphere had thus entered into the OPC stage.

### NSC-Induced OPCs can Differentiate into OLs

**Fig. 3:**
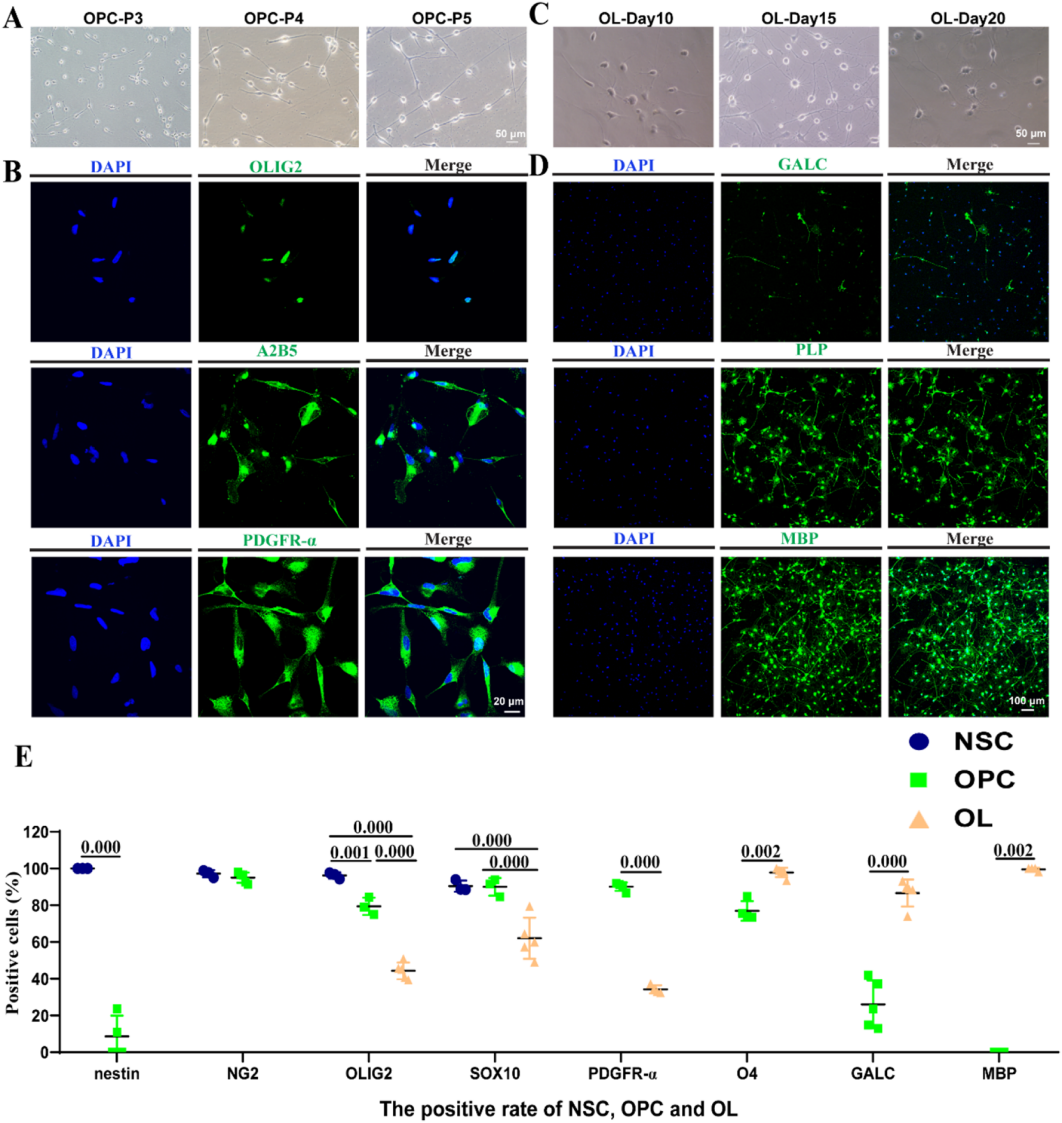
Verification of NSC-induced OPCs and differentiated OLs. **a** Morphologies of OPCs in passages 3–5 as observed; scale bar = 50 μm. **b** Protein expressions of OLIG2, A2B5, and PDGFRA in OPCs as confirmed by immunofluorescence assay; scale bar = 20 μm. **c** Morphologies of OLs on days 10, 15, and 21 as observed; scale bar = 50 μm. **d** Protein expressions of GALC, PLP, and MBP in OLs (21 days) as confirmed by immunofluorescence assay; scale bar = 100 μm. **e** Proportions of nestin^+^, NG2^+^, OLIG2^+^ cells, SOX10^+^ cells, PDGFR-α^+^ cells, O4^+^ cells, GALC^+^ cells and MBP^+^ cells analyzed by immunofluorescence staining. Statistical analyses of relative protein level in NSCs, OPCs and OLs. Means ± SD from three independent experiments represented. Nestin was highly expressed in NSCs, with a positive rate of 100%; when differentiated into OPCs, the positive rate of nestin decreased to 8.66%. NG2 was highly expressed in NSC and OPC, and the positive rates were 97.20% and 95.04%, respectively. OLIG2 and SOX10 were always expressed in OL lineage. The positive rates of OLIG2 in NSC, OPC, and OL were 96.26%, 79.46% and 44.38%, respectively, and the positive rates of SOX10 in NSC, OPC, and OL were 90.42%, 90.04% and 62.03%, respectively. PDGFR-α is one of the typical markers of OPCs. It is highly expressed in OPCs, and the positive rate was 90.15%. With the process of oligodendrocyte differentiation, the expression of PDGFR-α decreases, while the expression of O4 and GALC increases. The positive rates of PDGFRA in OPC and early OL were 90.15% and 34.26%. The positive rates of O4 in OPC and OL were 76.93% and 97.86%, respectively. The positive rates of GALC in OPC and early OL were 26.08% and 86.69%, respectively. When OPC differentiates into mature OL, MBP is highly expressed, with a positive rate of 99.55%, however, it is not expressed in OPC. For data that met the normality test, the positive rate was shown as the mean, otherwise it is shown as the median. Exact p-values have been reported

Here, NSCs differentiated into OPCs after 7 days of culturing. The morphological features of the OL lineage were as follows: OPC cell bodies were round or spindle-shaped, and their protrusions were bipolar and bead-like; elongated; or multipolar (Fig. 3a). Immunofluorescence staining of the OPCs showed that, aside from the expression of NG2 (an early marker of OPCs, mean: 95.04%) (Fig. 2c), A2B5 and PDGFR-α (mean: 90.15%) were also expressed. OLIG2 (mean: 79.46%), which was active throughout OL lineage, was also expressed (Fig. 3b). After 10 days of differentiation, the protrusions of the beaded bipolar cells gradually increased, multi-level protrusions began to branch (Fig. 3c), and GALC (mean: 89.69%) expression was observed (Fig. 3d). After approximately 20 days of differentiation, the protrusions increased, and membranes slowly formed (Fig. 3c). Immunofluorescence staining of OLs at this stage revealed that they were positive for PLP and MBP (Fig. 3d), and thus considered mature. Immunofluorescence staining revealed that nestin was highly expressed in NSCs, with a positive rate of 100%; once differentiated into OPCs, the positive rate of nestin decreased to 8.66%. OLIG2 and SOX10 were always expressed in the OL lineage. The positive rates of OLIG2 in NSC, OPC, and OL were 96.26%, 79.46%, and 44.38%, respectively, and the positive rates of SOX10 in NSC, OPC, and OL were 90.42%, 90.04%, and 62.03%, respectively. NG2 was highly expressed in NSC and OPC, with positive rates of 97.20% and 95.04%, respectively. PDGFR-α is a typical marker of OPCs. It was highly expressed in OPCs, and the positive rate was 90.15%. With the process of oligodendrocyte differentiation, the expression of PDGFR-α decreases, while the expression of O4 and GALC increases. The positive rates of PDGFR-α in OPCs and early OLs were 90.15% and 34.26%, respectively. The positive rates of O4 in OPCs and OLs were 76.93% and 97.86%, respectively. The positive rates of GALC in OPCs and early OLs were 26.08% and 86.69%, respectively. When OPCs differentiated into mature OL, MBP was highly expressed, with a positive rate of 99.55%, however, it was not expressed in OPC (Fig. 3e). For data that met the normality test, the positive rate was shown as the mean, otherwise it is shown as the median.

### Marker Changes During Differentiation of NSCs into OLs

**Fig. 4:**
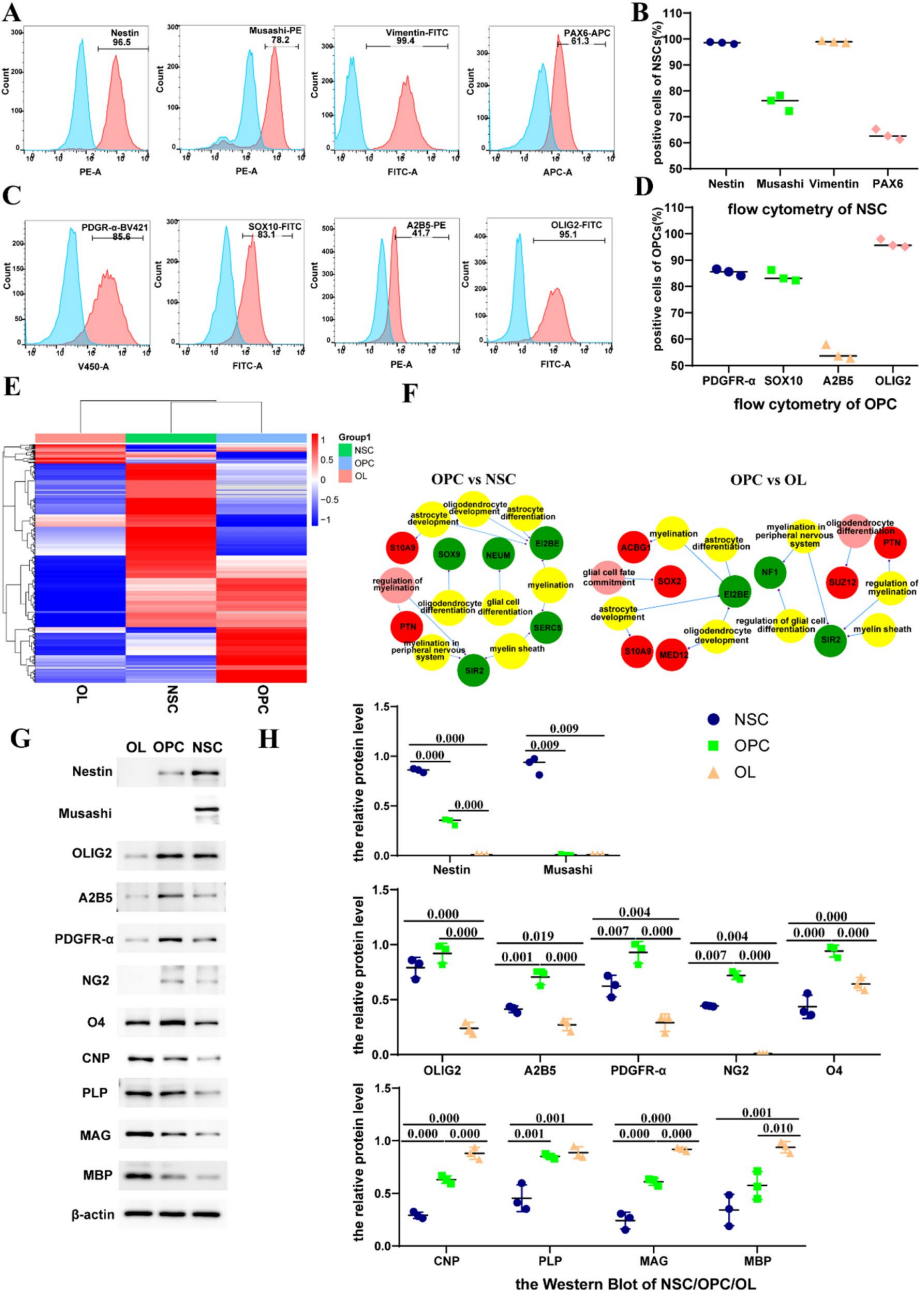
Marker changes during differentiation of NSCs into OLs. **a** Proportions of nestin^+^, Musashi^+^, vimentin^+^, and PAX6^+^ NSCs after culture day 6 as analyzed by flow cytometry. **c** Proportions of PDGFRA^+^, SOX10^+^, A2B5^+^, and OLIG2^+^ OPCs in passage 6 cultured cells as analyzed by flow cytometry. **b**–**d** Proportions of NSCs and OPCs; columns represent means ± SD. **e** Heatmaps of proteins in NSCs, OPCs, and OLs. **f** Interaction network of DE proteins and GO enrichments. **g** Representative Western blots and **h** Statistical analyses of relative protein level in NSCs, OPCs and OLs. For three samples, they met the homogeneity of variances, LSD test for post hoc multiple comparison were performed. Means ± SD from three independent experiments represented

The results of flow cytometry revealed that the proportion of cells positive for NSC cell markers, nestin, Musashi, vimentin, and PAX6, were 96.93%, 75.6%, 98.96%, and 63.06%, respectively (Fig. 4a, b); those for the OPC cell markers PDGFR-α, SOX10, A2B5, and OLIG2 were 85.43%, 83.86%, 54.8%, and 96.23%, respectively (Fig. 4c, d). Heatmaps of protein expression showed clear differences between the NSCs, OPCs, and OLs (Fig. 4e). Mapping of the different proteins and enriched GO terms in an interaction network (Fig. 4f) showed that *SOX9* and *NEUM*, which are related to oligodendrocyte and glial cell differentiation, respectively. *SOX9* were downregulated (≥ log2[fold change]) in OPCs compared to NSCs, probably because in the absence of SOX9, cells adopt a less mature state rather than progressing to a fully multipotent NSC, and did not give rise to astrocytes, oligodendrocytes, and neurons while adopt an early neuronal fate (Scott et al. 2010). *EI2BE*, which participates in oligodendrocyte development, astrocyte differentiation, and myelination, was downregulated in OPCs relative to OLs and OPCs relative to NSCs (≥ log2[fold change], Fig. 4f). NSCs can differentiate into astrocytes, EI2BE is involved in astrocyte differentiation and development which explains that EI2BE was down regulated in OPCs compared to NSCs. Western blot results for NSCs, OPCs, and OLs showed that nestin and Musashi were highly expressed in NSCs, expression of OLIG2, A2B5, PDGFR-α, NG2, and O4 was higher in OPCs than in NSCs and OLs, and the expression of CNP, MAG, and MBP was highest in OLs, followed by OPCs, and lowest in NSCs (Fig. 4g, h). For data that met the normality test, the positive rate was shown as the mean, otherwise it is shown as the median.

### Expression of mRNAs by RNA-seq and qPCR Analysis

**Fig. 5:**
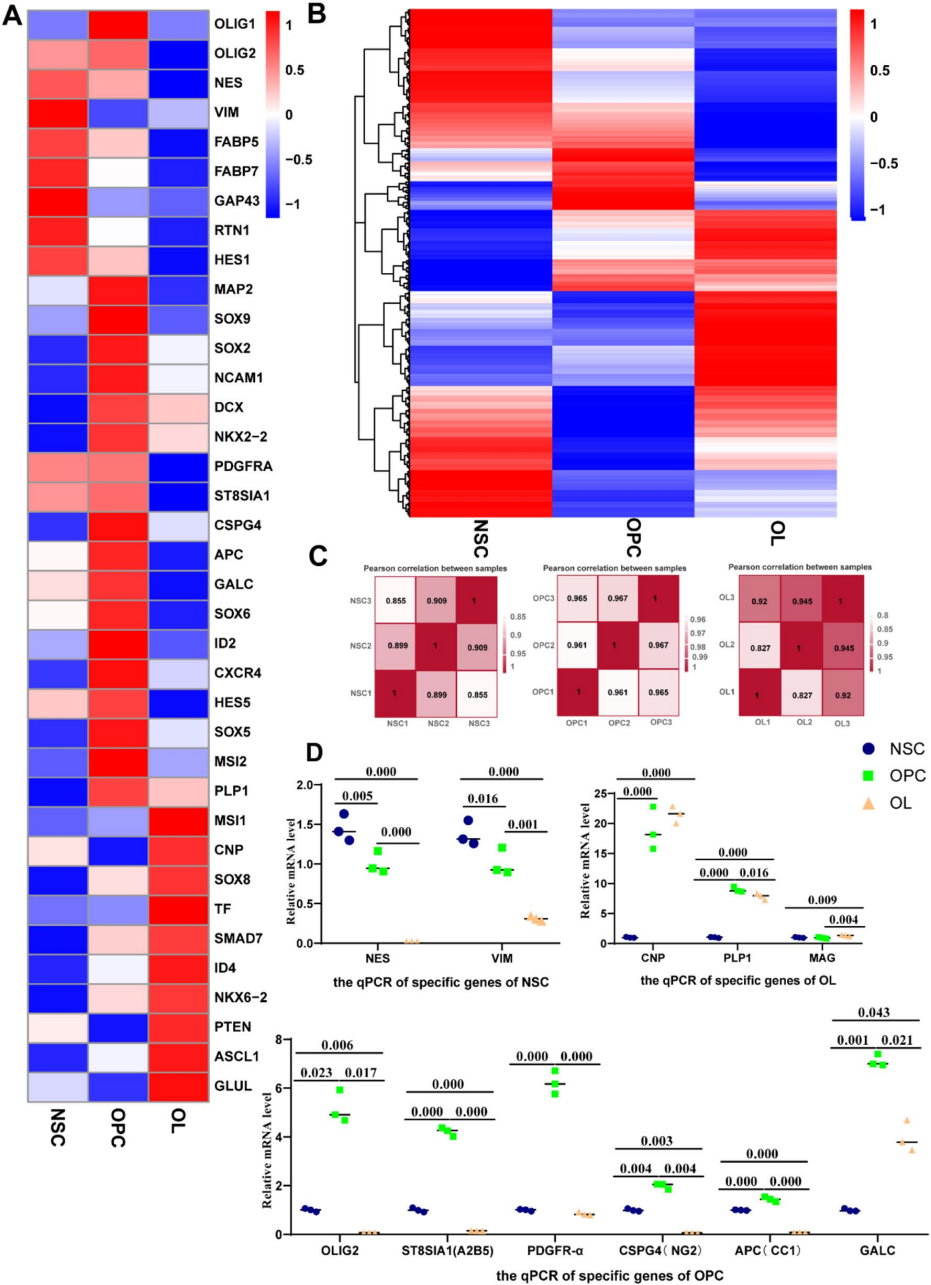
Marker changes during differentiation of NSCs into OLs. **a** Heatmap of genes, related to differentiation and myelination, expressed in NSCs, OPCs, and OLs. **b** Heatmap of total mRNAs in NSCs, OPCs, and OLs. **c** Spearman’s rank correlation of RNA-seq for three samples. **d** mRNA levels of NSC, OPC, and OL markers as detected by qPCR. Means ± SD from three independent experiments represented; .For three samples, they met the homogeneity of variances, LSD test for post hoc multiple comparison were performed; *P < 0.05; **P < 0.01; ***P < 0.001

We constructed heatmaps of fully expressed mRNAs for NSCs, OPCs, and OLs (Fig. 5a) as well as mRNAs related to the differentiation and maturation of NSCs into OLs (Fig. 5b). RNA-seq results revealed that *VIM*, *NES*, *FABP7*, *FABP5*, and *HES1* were highly expressed in NSCs (Fig. 5a). The expressions of *VIM* and *NES* corresponded with the qPCR results (Fig. 5d). *SOX9*, *SOX2*, *NKX2-2*, *PDGFRA*, *ST8SIA1* (protein: A2B5), *CSPG4* (protein: NG2), and *APC* (protein: CC1) were highly expressed in OPCs, as shown in RNA-seq results (Fig. 5a), and the expressions of *NKX2-2*, *PDGFRA*, *ST8SIA1*, *CSPG4*, and *APCs* were consistent with the qPCR results (Fig. 5d). In OLs, *CNP*, *NKX6-2*, *ID4*, *PTEN*, and *ASCL1* were highly expressed, according to RNA-seq results (Fig. 5a). The expressions of *CNP* and *PLP1* were consistent with the qPCR results (Fig. 5d). Although the expression of *MAG* was not detected by RNA-seq, qPCR verified its expression (Fig. 5d); the expression of *MBP* was also confirmed by western blot and immunofluorescence staining (Figs. 3d, 4d). These findings demonstrate the characteristics of the NSCs, OPCs, and OLs generated in this study. Furthermore, correlation analysis of each sample in the NSC, OPC, and OL groups revealed only small intragroup differences (Fig. 5c).

### DE Genes and Their GO Enrichment

**Fig. 6:**
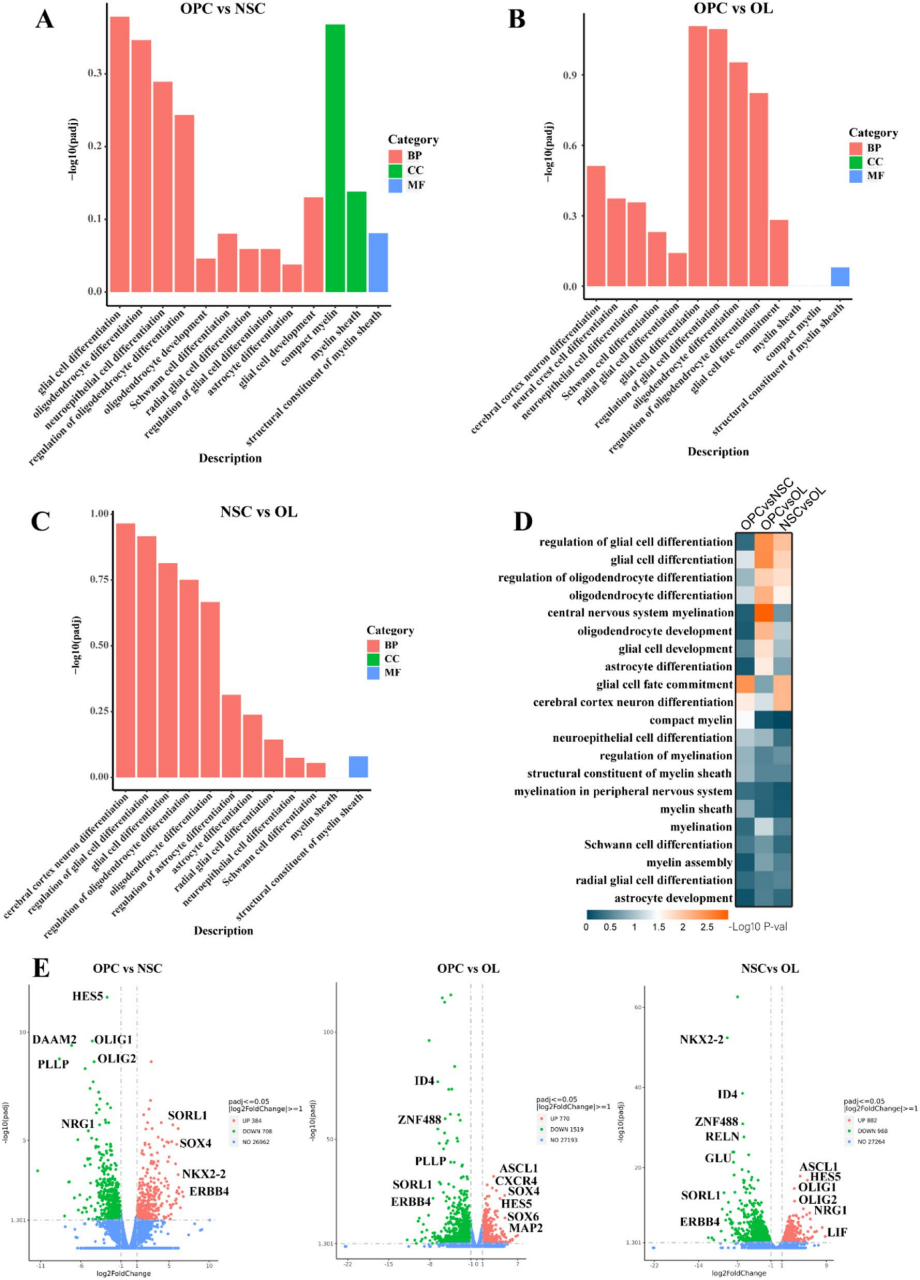
DE genes and GO enrichments. **a**–**c** GO enrichment terms for DE mRNAs related to differentiation and myelination in comparisons of OPCs vs. NSCs, OPCs vs. OLs, and NSCs vs. OLs, respectively. The abscissa was GO terms, and the ordinate was − log10 (*P* value). **d** GO enrichment terms for DE mRNAs related to differentiation and myelination in comparisons of OPCs vs. NSCs, OPCs vs. OLs, and NSCs vs. OLs, respectively, with − log10 *P* values. **e** DE mRNAs associated with differentiation and myelination

To identify the biological processes related to the DE mRNAs of NSCs, OPCs, and OLs, we performed GO enrichment analysis (log2[fold change] ≥ 1) for DE mRNAs between OPCs and NSCs, OPCs and OLs, and NSCs and OLs. The GO functions of the differential gene enrichment of OPCs and NSCs were as follows: biological process (BP; glial cell differentiation, oligodendrocyte differentiation, neuroepithelial cell differentiation, OL development, and glial cell development); cellular component (CC; compact myelin and myelin sheath); and molecular function (MF; structural constituent of myelin sheath) (Fig. 6a–d). When comparing OPCs and OLs, the enriched differential gene functions concerned the development and differentiation of glial cells and OLs. When comparing NSCs and OLs, the enriched differential gene functions were related to the development of radial glial cells, neurons, and astrocyte cells (Fig. 6a–d). We investigated the expression profiles of mRNAs in NSCs, OPCs, and OLs using RNA-seq. When comparing OPCs to NSCs, we found that 384 mRNAs were upregulated, and 708 mRNAs were downregulated significantly. When comparing OPCs to OLs, we found that 770 mRNAs were upregulated, while 1519 mRNAs were significantly downregulated. We also showed that some genes may be related to differentiation and myelination using volcanic plots (Fig. 6e).

### DE Genes and Their KEGG Pathways

**Fig. 7:**
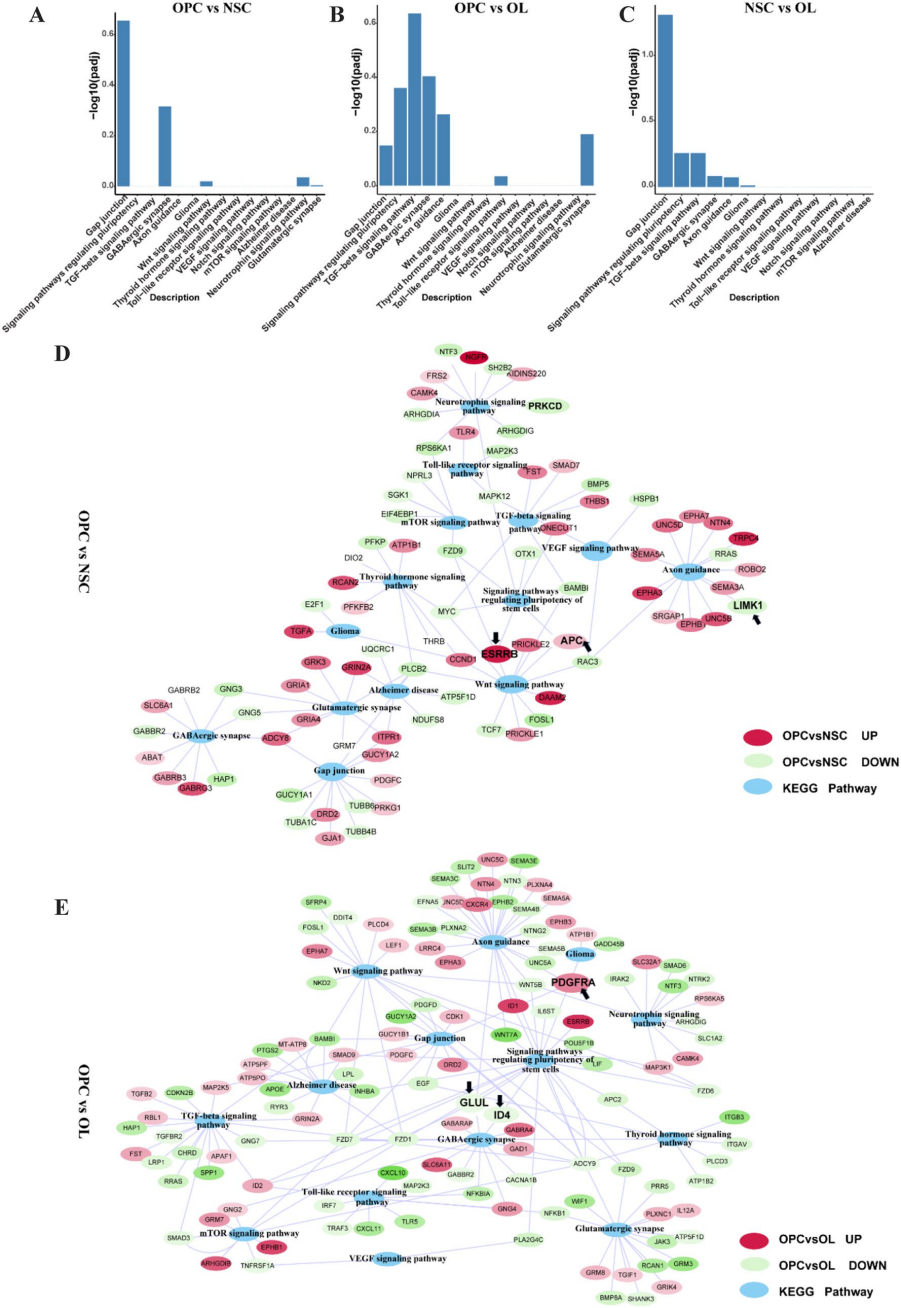
DE genes and KEGG pathways. **a**–**c** KEGG pathway terms for DE mRNAs related to differentiation and myelination in comparisons of OPCs vs. NSCs, OPCs vs. OLs, and NSCs vs. OLs, respectively. The abscissa was KEGG terms, and the ordinates was − log10 (*P* value). **d**, **e** Interaction network for DE mRNAs and KEGG pathway terms in comparisons of OPCs vs. NSCs and OPCs vs. OLs, respectively. Blue ovals represent KEGG pathway terms; red ovals for upregulated mRNAs; green ovals represent downregulated mRNAs. Functions that may influence NSC-OL differentiation are shown in bold black font

We performed KEGG pathway enrichment analysis to identify pathways associated with DE mRNAs between NSCs, OPCs, and OLs (Fig. 7a–c). The pathways identified included gap junctions, signaling pathways regulating the pluripotency of stem cells, transforming growth factor (TGF)-β signaling, γ-aminobutyric acid (GABA)-ergic synapse, axon guidance, glioma, Wnt signaling, thyroid hormone signaling, toll-like receptor signaling, vascular endothelial growth factor (VEGF) signaling, Notch signaling, mammalian target of rapamycin (mTOR) signaling, Alzheimer’s disease, neurotrophin signaling, and glutamatergic synapse. We mapped the different mRNAs and enriched KEGG pathways into one interaction network (Fig. 7d, e) and found that the mRNAs of different pathways overlapped. KEGG enrichment analysis revealed, for instance, that the Wnt signaling pathway, *APC* and *ESRRB* (related to oligodendrocyte and stem cell differentiation) were upregulated (log2[fold change] ≥ 1) in OPCs compared to NSCs. Moreover, *LIMK1*, which participates in axon extension and the nervous system development, was downregulated in OPCs relative to NSCs (log2[fold change] ≥ 1) (Fig. 7d). *PDGFRA*, which affects the differentiation and proliferation of OPCs, was upregulated (log2[fold change] ≥ 1) in OPCs, compared to OLs. Finally, *ID4* and *GLUL*, which are related to myelination in OLs, were downregulated in OPCs relative to OLs (Fig. 7e).

### Potential Influence of mRNAs on the Differentiation of NSCs and Maturation of OLs

**Fig. 8:**
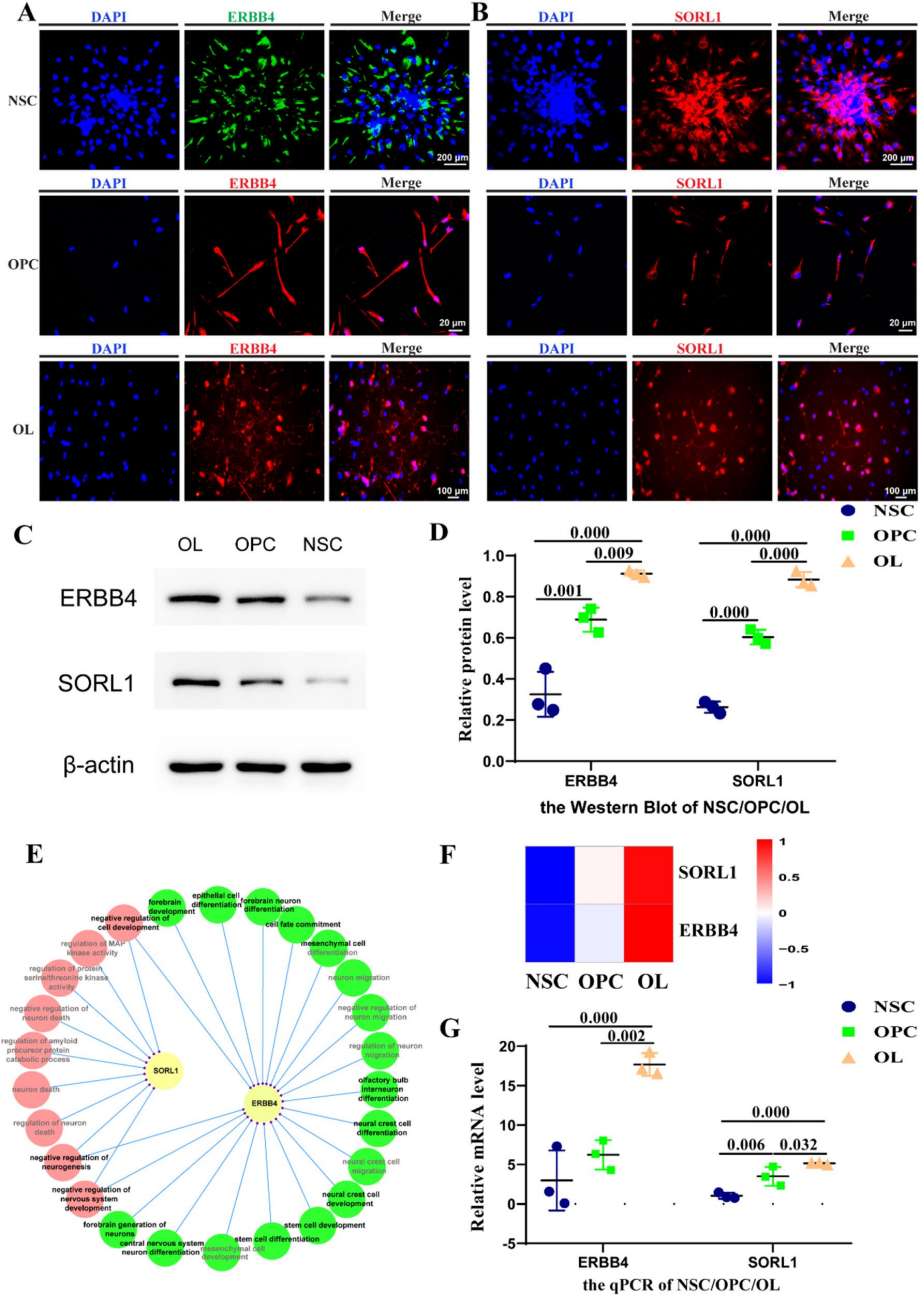
ERBB4, SORL1, and their GO enrichment. **a** Protein expressions of ERBB4 in NSCs, OPCs, and OLs as confirmed by immunofluorescence assay; scale bar = 200 μm, 20 μm, and 100 μm. **b** Protein expressions of SORL1 in NSCs, OPCs, and OLs as confirmed by immunofluorescence assay; scale bar = 200 μm, 20 μm, and 100 μm. **c** Representative Western blots and **d** Statistical analyses of ERBB4 and SORL1 of relative protein level in NSCs, OPCs and OLs. Means ± SD from three independent experiments represented. **e** Interaction network of ERBB4, SORL1, and GO enrichment terms that may be related to differentiation and myelination, ERBB4 and SORL1 may affect NSC differentiation and OL maturation through olfactory bulb interneurons and by regulating the development of the CNS, respectively. **f** Heatmap of *ERBB4 and SORL1* expressed in NSCs, OPCs, and OL. **g** *ERBB4 and SORL1* levels of NSC, OPC, and OL markers as detected by qPCR. Means ± SD from three independent experiments represented. *ERBB4 and SORL1* gradually increased during NSC-OL differentiation

The analysis of DE mRNAs of OPCs compared to NSCs and OLs, respectively, showed that *ERBB4* and *SORL1* were upregulated significantly, at thresholds for *P*-adj ≤ 0.05 and log2[fold change] ≥ 1 during NSC-OL differentiation, shown by the heatmap (Fig. 8f) and volcanic plots (Fig. 6e). The fragments per kilobase of transcript per million mapped reads (FPKM) of *ERBB4* was the highest in OLs, followed by OPCs, and was lowest in NSCs (*P*-adj < 0.05, log2[fold change] > 1) (Figs. 6e, 8f). The GO terms that were enriched by *ERBB4* revealed that it was related to the development of the CNS (Supplemental Fig. 1a–d). A similar trend was observed for *SORL1*, which was identified as a DE mRNA when comparing OPCs with NSCs and OPCs with OLs (Supplemental Fig. 1a–d). *ERBB4* and *SORL1* were involved in GO terms related to development, stem cell differentiation, and nervous system development (Supplemental Fig. 1a–d, Fig. 8e). Moreover, the interaction network revealed an overlap between the DE mRNAs in GO functions (Fig. 8e). Shown by immunofluorescence staining, ERBB4 and SORL1 were expressed in NSCs, OPCs, and OLs (Fig. 8a, b). Western blot results for ERBB4 and SORL1 in NSCs, OPCs, and OLs showed that the expression of ERBB4 and SORL1 were the highest in OLs, followed by OPCs, and lastly NSCs (Fig. 4c, d). We also detected the mRNA expression of ERBB4 and SORL1 by qPCR and RNA-seq in NSC, OPC and OL, and the results showed that their expression gradually increased with the progress of differentiation (Fig. 4f, g).

## Discussion

Following recent advances in genomics, the role of RNAs in biological and developmental processes has elucidated the complexity of the human genome. Moreover, new approaches for studying stem cell development are continuously being developed. The extensive research of mRNAs has gradually revealed their involvement in NSC differentiation and OL myelination (Yu et al. 2013; Flagelli et al. 2021). It is hypothesized that mRNAs regulate stem cell development through various mechanisms (Liu et al. 2020). However, few comprehensive methods exist for analyzing mRNAs in their regulation of differentiation of NSC and maturation of OL. Here, the expression profiles of mRNAs in NSCs, OPCs, and OLs were investigated using RNA-seq to explore the functions and interactions of mRNAs.

To this end, we aimed to establish an efficient strategy for generating OPCs. As previously mentioned, conventional sources of OPCs include hESCs, hiPSCs, and human fetal brain tissue (Dietz et al. 2016; Nandakumar et al. 2021; Azari 2022). Human blastocyst inner cell mass-derived hESCs can be cultured on a large scale and give rise to large numbers of transplantable precursor cells, though the ethical issues around the sourcing of hESCs complicate their use. Unlike hESCs, there are no ethical concerns regarding the sourcing of hiPSCs, which are derived from relatively easily obtained somatic cells, such as fibroblasts (Chanoumidou et al. 2020). However, the generation of OPCs from hESCs and hiPSCs is time-consuming, residual undifferentiated cells can form tumors, and the gene editing technology suffers from genetic instability; these issues complicate the clinical application of hESCs and hiPSCs (Wang et al. 2013). As early as 1999, Roy et al. isolated and extracted OPCs from adult brain tissue using flow sorting (Roy et al. 1999). However, brain tissue samples are difficult to source, and OPCs only account for 3% of adult brain tissue cells; thus, the clinical application of human brain tissue-derived OPCs is limited (Abdolahi et al. 2021). In comparison, human NSCs are relatively easy to obtain and can be induced to differentiate into various neural cells, including OLs, astrocytes, and neurons. With the action of mitogens, NSCs have unlimited self-renewal and proliferation capabilities, and a large number of high-purity OPCs can be obtained in a short time. Additionally, the risk of tumorigenicity is low, which simplifies their translation into clinical research (Lu et al. 2015).

iPSCs induce oligodendrocytes for a longer time. Within 8 days, iPSCs differentiate into PAX6^+^ NSCs, which give rise to OLIG2^+^ progenitors by day 12. OLIG2^+^cells begin to express NKX2.2 around day 18, followed by SOX10 around day 40. OPCs that are positive for O4 antibody appear around day 50 and reach. On average, 43% of the cell population after 75 days of differentiation, OPCs can be terminally differentiated to MBP^+^ OLs (Douvaras and Fossati 2015). hESCs can be differentiated into OPCs. OPCs after 9 weeks of differentiation expressed OLIG2, SOX10, and NKX2.2 at elevated levels. According to the flow cytometric analysis, the cells expressed A2B5 (> 70%) and NG2 (40–60%) at 5-week time point, whereas maturing oligodendrocytes expressed O4 (60–80%) at 11-week time point (Sundberg et al. 2011). While the hOPCs in our study, the cells expressed A2B5 (> 50%), PDGFR-α (80–90%), OLIG2 (> 90%), and SOX10 (80–90%) at 1-week time point. The induction time of NSCs into OLIG2^+^ and SOX10^+^ OPCs (80–90%) took only 7 days, 5 days less than OLIG2^+^ hOPCs, 33d less than SOX10^+^ hOPCs induced by hiPSCs. The induction time of OLIG2^+^ and SOX10^+^ hOPCs induced by NSCs was 54 days less than that by hESCs. The induction time of A2B5^+^ and NG2^+^ hOPCs was 28 days shorter than that of A2B5^+^ and NG2^+^ hOPCs induced by hESCs. In addition, our OPCs also expressed PDGFR-α. It took only 10 days for our OPCs to differentiate into GalC^+^ OLs, and 21 days for OPCs to differentiate into MBP^+^ OLs (> 90%). The differentiation time of MBP^+^ OLs (43%) was 24 days less than that of hOPCs induced by hiPSCs. In this study, there is a time advantage the process of NSC-induced OPCs and OPCs-differentiated OLs. The specific protein expression level was high. We established efficient directional differentiation method for obtaining high-quality of OPCs and OLs from NSCs. We not only shorten the culture span by the current method, but alao optimize our induction methods. Our induction method additionally adds transferrin, Heparin, Lactate, and LIF compared with the previous induction methods of ESC and iPSC. OPCs with transferrin (Tf) leads to Fyn kinase activation which has multiple functions during OL differentiation and during myelination by the mechanism that involves transferrin receptor. The stimulatory effect of Tf on the maturation of OPCs is regulated by Fyn/MEK/ERK signaling as well as by PI3K/Akt signaling. Moreover, transferrin receptor is fundamental for signal transduction (Pérez et al. 2013). Since increasing OPC yields were concerned, we used Heparin to improve cell biomass yields. Addition of Heparin produced higher numbers of NG2 cells and generated more MBP mature OL after differentiation (Franco et al. 2015). Lactate directly promotes differentiation and cell cycling in the culture of OPC. The metabolite of glycogen, lactate, contribute to differentiation of OPCs and cell cycling. Lactate may contribute to promoting recovery for instance by remyelination in demyelinated tissue in the CNS (Ichihara et al. 2017). LIF is well known to promote the differentiation of OPCs and the maturation of OLs to myelination and remyelination to repair myelin (Rittchen et al. 2015). Transferrin, Heparin, Lactate, and LIF were added additionally to the OPCs induction medium, which maybe the reason for more effective and rapid induction to OPCs derived from NSCs than previous reported method.

The seed cells in our study are neural stem cell lines which has recently been authenticated and by National Institutes for Food and Drug Control of China (Report Number: SH20220032). We have established a huge system which can be passaged more than 60 times and cultured as floating neuro-spheres with a proliferation factor of 3–4 times in every passage. These advantages make it easy to obtain NSCs in our research. The NSC system provides an adequate source for OPC induction and OLs differentiation. OPC were incubated on 6-well plates at a density of 200,000 cells/well or on a T75 cm^2^ plate at a density of 20,000 cells/cm^2^ with a proliferation factor of 3–5 times in every passage. The yield of OPCs is sufficient to meet the amount of cells needed for clinical transplantation in the National Key Research and Development Program of China (2017YFA0104200) and meet our research. For the culture of OLs, although they cannot be passed down, after 21 days of OLs cultured with 6-well plates at a density of 200,000 cells/well, the quantity of OLs can met experiments of our research. Based on a huge system of NSCs easily obtained cultured on T75 and even T225, OPCs with large amplification, and OL cultured on 6-well plates or even T75, we have established method for obtaining large quantities of human OL progenitor cells (OPCs) and OLs from NSCs. The yields of our NSCs, OPCs, and OLs meet the needs of our research and clinical transplantation.

We also analyzed GO enrichment and KEGG pathways of DE mRNAs to determine their associated pathways and molecular functions. By comparing OPCs to NSCs and OLs, respectively, we found that the GO enrichment functions related to the differentiation of glial cells and OLs (BP), compact myelin and the myelin sheath (CC), and the structural constituents of the myelin sheath (MF) varied significantly in both comparisons. However, when compared under the same conditions, the functional differences between OPCs and NSCs mainly reflected the fate of glial cells, while the differences between OPCs and OLs were mainly related to the differentiation of glial cells and OLs, and mature of OL in the CNS. GO enrichment analysis demonstrated that, when comparing OPCs with NSCs, the upregulation of NKX2-2 in OPCs promoted the differentiation of NSCs to OPCs, while NGR1 maintained the stemness of NSCs. When comparing OPCs and OLs, the upregulation of ID4 and PLLP in OLs may have contributed to their maturation. These increases in ID4 and RELIN in OLs were also consistent with their expression in the mature state, when comparing NSCs and OLs. We already know that mRNAs associated with the differentiation of NSCs to OLs may alter the gene expression of *OLIG1* (Zhao et al. 2022), *OLIG2* (Yu et al. 2013), *NKX2-2* (Yuan et al. 2022), *SOX4* (Braccioli et al. 2018), *PLLP* (Shulgin et al. 2021), *ZNF488* (Wang et al. 2006), *ID4* (Huang et al. 2022), and *RELN* (Liang et al. 2019). According to the literature, gap junctions (Fasciani et al. 2018), signaling pathways regulating the pluripotency of stem cells, TGF-β signaling (Kahm et al. 2021), Wnt signaling (Fang et al. 2021; Zheng et al. 2019), thyroid hormone signaling (Lee and Petratos 2016), VEGF signaling (Cervenka et al. 2020), Notch signaling (Zheng et al. 2021; Tiane et al. 2021), mTOR signaling (Liu et al. 2022; Shao et al. 2021), and glutamatergic synapse (Bergles et al. 2000) were related to the mature of oligodendrocyte lines. KEGG pathway analysis on the differential mRNAs of NSCs, OPCs, and OLs confirmed that the same pathways affected mature of oligodendrocyte lines; when comparing OPCs to OLs, the difference was significant. In addition, the network diagram of mRNAs and pathways showed that *PDGFRA* (Hamashima et al. 2020), *APC* (Bin et al. 2016; Ulloa-Navas et al. 2021), *GLUL* (Ueki et al. 2015), *ID4* (Tiane et al. 2021; Huang et al. 2022), and other mRNAs may be important in the regulation of myelination.

In this study, we described a method for screening key mRNAs that may regulate myelination. ERBB4 is a member of the epidermal growth factor receptor subfamily and the Tyr protein kinase family. This protein is activated by and binds to neuregulin and other factors. It induces diverse cellular responses, including differentiation and mitogenesis (Zimonjic et al. 1995). ERBB4 is also involved in the development of the nervous system (Lin et al. 2018) and multicellular organisms (Gaudet et al. 2011), as well as the migration of neural crest cells (Hyder et al. 2021) and the differentiation of olfactory bulb interneurons (Marin and Rubenstein 2003). It may also be involved in amyotrophic lateral sclerosis (Sun et al. 2020), peripheral nerve sheath tumors (Longo et al. 2019), Alzheimer’s disease (Zhang et al. 2021), Parkinson’s disease (Depboylu et al. 2012), and glioblastoma (Jones et al. 2018), among other diseases. Interestingly, ERBB4 signaling has been known to mediate the survival of pre-neurons (Plani-Lam et al. 2015). We hypothesize that ERBB4 may affect NSC differentiation and OL maturation through olfactory bulb interneurons and by regulating the development of the CNS, respectively. ERBB4 may regulate the differentiation of intermediate precursor neurons into OLs (Berg et al. 2018; Ma et al. 2018; Kriegstein and Alvarez-Buylla 2009), that is, the process by which NSCs differentiate into OLs may involve the interneuron precursor neuron stage (Kriegstein and Alvarez-Buylla 2009). We found that SORL1 belongs to at least two families: the low-density lipoprotein receptors (LDLR) and the vacuolar protein sorting 10 (VPS10) domain-containing receptors (Wang et al. 2022). Although it does not appear related to the maturation and differentiation of OLs, we found that this gene was associated with beta-amyloidosis (Knupp et al. 2020) and Alzheimer’s disease (Mishra et al. 2022). More importantly, we found that SORL1 regulates the secretion of glial cell line-derived neurotrophic factors and that SORL1 mediates tropomyosin receptor kinase B trafficking, thus enhancing neuronal responses to BDNF (Rohe et al. 2013). Therefore, SORL1-influenced neurotrophic factors and BDNF (Eggert et al. 2022) may be responsible for the differentiation and maturation of OLs.

## Conclusion

NSCs can differentiate into neurons, astrocytes, and OLs efficiently. By analyzing the DE mRNAs and proteins of NSCs, OPCs, and OLs, we were able to identify PDGFR-α, APC, ID4, PLLP, and other markers related to NSC differentiation and OL maturation. Moreover, we refined a screening method for ERBB4 and SORL1 that may underlie regulate NSC differentiation and OL maturation. In future research, animal disease or gene knockout models should be used to confirm the in vivo functions of the mRNAs highlighted in our study. In addition, in vitro gene editing should allow for the direct observation of NSC differentiation and OL maturation.

## Supplementary Information

Below is the link to the electronic supplementary material.

Supplementary file1 (DOCX 653 kb)

## Data Availability

The datasets generated during and/or analyzed during the current study are not publicly available, but are available from the corresponding author on reasonable request.
