# Supplementary material for: Identifying Genes that Affect Differentiation of Human Neural Stem Cells and Myelination of Mature Oligodendrocytes
Source: Cell Mol Neurobiol. 2022 Dec 22;43(5):2337–58. doi: 10.1007/s10571-022-01313-5 (PMC10287785; doi:10.1007/s10571-022-01313-5)
Supplement: Supplementary file 1 — Supplementary file1 (DOCX 653 kb) [file 10571_2022_1313_MOESM1_ESM.docx]

Identifying genes that affect differentiation of human neural stem cells and myelination of oligodendrocytes

Dou Ye, Qian Wang, Yinxiang Yang, Bingyu Chen, Fan Zhang, Zhaoyan Wang, Zuo Luan

**Supplemental Table 1. List of matierials and regeants of** **the culture of OPCs**

| **Matierials and regeants** | **companies** | **catalog numbers** |
| --- | --- | --- |
| **Neurobasal™-A Medium** | **Gibco** | **10888-022** |
| **DMEM** | **Gibco** | **C11330500BT** |
| **putrescine** | **Sigma** | **P5780** |
| **transferrin** | **Sigma** | **T8158** |
| **GlutaMAX™-1** | **Gibco** | **35050-061** |
| **B27** | **Gibco** | **17504-044** |
| **PDGF** | **Pepro Tech Inc** | **AF-100-13A** |
| **NT3** | **Pepro Tech Inc** | **AF-450-03** |
| **bFGF** | **Pepro Tech Inc** | **100-18C** |
| **EGF** | **Pepro Tech Inc** | **AF-100-15** |
| **LIF** | **Pepro Tech Inc** | **300-05** |
| **trypsin** | **Gibco** | **15050-065** |
| **trypsin inhibitor** | **Sigma** | **T6522** |
| **penicillin** | **Sigma** | **P3032** |
| **streptomycin** | **Sigma** | **S9137** |
| **PBS** | **Gibco** | **10010023** |
| **Laminin** | **Invitrogen** | **23017-015** |

**Supplemental Table 2. List of matierials and regeants of the culture of OLs**

| **Matierials and regeants** | **companies** | **catalog numbers** |
| --- | --- | --- |
| **Oligodendrocyte Precursor Cell Differentiation Medium** | **Sciencell Resarech Laboratories** | **1631** |
| **DMEM** | **Gibco** | **C11330500BT** |
| **DAPT** | **Sigma** | **D5942** |
| **PBS** | **Gibco** | **10010023** |
| **penicillin** | **Sigma** | **P3032** |
| **streptomycin** | **Sigma** | **S9137** |
| **Poly-L-ornithine hydrobromide** | **Sigma** | **P3655** |
| **Laminin** | **Invitrogen** | **23017-015** |

**Supplemental Table 3. List of matierials and regeants of the western blot**

| **Matierials and regeants** | **companies** | **catalog numbers** | **RRID** |
| --- | --- | --- | --- |
| **Nestin** | **Abcam** | **ab6320** | **AB_308832** |
| **Musashi** | **Abcam** | **ab52865** | **AB_881168** |
| **OLIG2** | **Millipore** | **AB9610** | **AB_570666** |
| **A2B5** | **Thermo Fisher Invitrogen** | **433110** | **AB_2532204** |
| **PDGFR-α** | **Abcam** | **ab32570** | **AB_777165** |
| **NG2** | **Abcam** | **ab83178** | **AB_10672215** |
| **O4** | **Biorbyt** | **orb664340** |  |
| **CNPase** | **Abcam** | **ab6319** | **AB_2082593** |
| **PLP** | **Abcam** | **ab9311** | **AB_2165790** |
| **MAG** | **Sigma-Aldrich** | **MAB1567** | **AB_2923508** |
| **MBP** | **Abcam** | **ab7349** | **AB_305869** |
| **ERBB4** | **Proteintech** | **19943-1-AP** | **AB_10646486** |
| **SORL1** | **Cell Signaling Technology** | **79322** | **AB_2799927** |
| **β-actin** | **Abcam** | **ab8226** | **AB_306371** |

**Supplemental Table 4. List of primers**

| Genes | Primer sequences (5′–3′)  Forward | Primer sequences (5′– 3′)  Reverse | Product length (bp) |
| --- | --- | --- | --- |
| NES | GAAACAGCCATAGAGGGCAAA | TGGTTTTCCAGAGTCTTCAGTGA | 167 |
| VIM | AGTCCACTGAGTACCGGAGAC | CATTTCACGCATCTGGCGTTC | 98 |
| OLIG2 | CCAGAGCCCGATGACCTTTTT | CACTGCCTCCTAGCTTGTCC | 178 |
| ST8SIA1 | TACTCTCTCTTCCCACAGG | GACAAAGGAGGGAGATTGC | 149 |
| PDGFRA | TGGCAGTACCCCATGTCTGAA | CCAAGACCGTCACAAAAAGGC | 88 |
| CSPG4 | GCCACGTTGTCAGTCGATG | CCCATAGGGGACCTCTAGGG | 75 |
| GALC | GCAACCTCCCGACTTCTAGTA | ACCACTCGTATCCTCGGAAATA | 199 |
| APC | GGTTGGCACTCTTACTTACC | TTCCACATGCATTACTGACTAT | 200 |
| CNP | AACAGAGGCTTCTCCCGAAAA | GTCTTGCACTCTAGCAGCGT | 149 |
| PLP1 | ACCTATGCCCTGACCGTTG | TGCTGGGGAAGGCAATAGACT | 109 |
| MAG | GGTGTCTGGTACTTCAATAGCC | CTCTCGTGGACTACTTGGGTG | 83 |
| ERBB4 | ACAATGTGACGGCAGATGCTACG | AGTCTGTGTCCTTAGGTCCTGAGC | 92 |
| SORL1 | CAAGGTGTACGGACAGGTTAGT | CCAATGCCAGGCTATCTCG | 113 |
| β-actin | ATCACCATTGGCAATGAGCG-3' | 5'-TTGAAGGTAGTTTCGTGGAT | 98 |

**
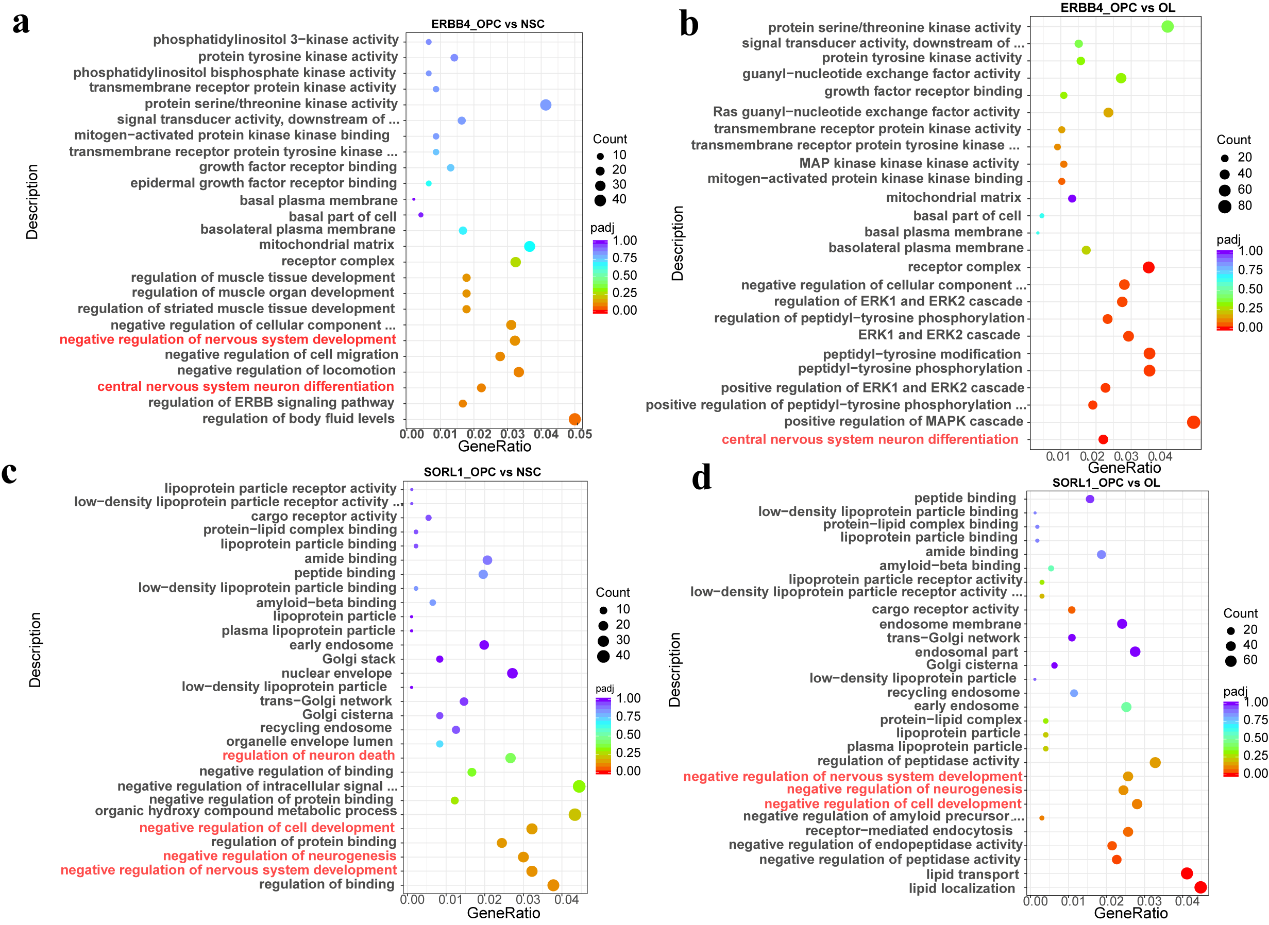
**

**Supplemental Fig.1. ERBB4, SORL1, and their GO enrichment.** A–D) Top GO enrichment terms for DE mRNAs, ERBB4 and SORL1, related to differentiation and myelination in comparisons of OPCs vs. NSCs and OPCs vs. OLs, respectively.
